# Editorial: Obstacles, advantages, and recent progress in honey bee virus research

**DOI:** 10.3389/finsc.2023.878344

**Published:** 2023-04-03

**Authors:** Wei-Fone Huang

**Affiliations:** College of Animal Science (College of Bee Science), Fujian Agriculture and Forestry University, Fuzhou, Fujian, China

**Keywords:** deformed wing virus, antiviral protein, quasi-species, honeybee, virus subtype

Exploring the unknown and conquering obstacles are always important for insect pathogen studies. The opportunities for exploiting new technologies and materials will always be tempting to researchers in this field. In this Research Topic, many scientists utilized new materials and methods to identify evidence that may lead to a further understanding of bee pathogens. Among the pathogens of interest, deformed wing virus (DWV) and its subtypes were the major viral pathogens addressed in this Research Topic. DWV is proposed to be a quasi-species (1), loosely consisting of subtypes that were previously considered as separated virus species. Subtype A was the original DWV, and subtype B was *Varroa destructor* virus-1 (VDV-1), named since the first identification was in *Varroa* mites (2). DWV subtypes A and B largely overlap in distribution and can co-infect the same hosts although the factors involved with these co-infections are not yet fully revealed. In addition to subtypes A and B, DWV quasi-species may contain subtype C and Kakugo virus, but these two subtypes have not been formally recognized. Kakugo virus was identified in the central neuron system of unusually aggressive bees in Japan (3). Kakugo virus shares 98% genome similarity with DWV-A but seems to enhance aggressive behaviors that have not been identified in DWV-A infections. These DWV subtypes have different regional distributions. Kakugo virus might have a limited distribution in East Asia and be better adapted to environments with both domestic European and Asian honey bees based on my preliminary survey. However, this preliminary observation was not fully supported by Zhang et al. as seen in their article published on this Research Topic. They analyzed four different genome fragments from DWV variants identified in European and Asian honey bees, wasps, and stingless bee taxa that were poorly addressed in previous studies. Another interesting research direction of the DWV quasi-species is how these subtypes compete within the same host and if Varroa mite has a specific role in the subtype competitions. Bai et al. in this Research Topic used long-read sequencing, also known as third-generation sequencing, to identify associations between DWV variants and Varroa mite haplotypes. They showed that DWV variants associated with mites tend to be regionally distributed. Penn et al. investigated the factors that could affect DWV subtypes within the same bee species, *Apis mellifera*, under different treatments. Interestingly, they found that creating a wound on the bees alone could significantly alter the infection dynamics between DWV-A and - B, and infection intensities were distributed differently in tissues.

Identifying novel anti-pathogenic molecules in honey bees is always an interesting challenge. Insects have a vast diversity, including in their responses to pathogens. Some common immune responses of insects, such as melanization cascade that involve phenol-oxidase and cellular immune responses that use phagocytosis, have been listed in textbooks. These responses were revealed in the earlier days by studying bacterial and fungal pathogens that can be visible under microscopes, leading to intuitive identifications of pathogens and host immune responses. In addition, the *in-vitro* cultures and consequent isolation of bacteria and fungi were well-developed and have been applied to studying the anti-microbial activities of insects. In contrast, these research tools were unavailable for virus-related immune responses, slowing research progress. However, antiviral responses in bees are critical in studies addressing virus diseases and their impacts on population decline. Moreover, insect antiviral immune responses are difficult to predict using gene sequences because of the high diversity in both hosts and viruses. Although the honey bee genome is relatively well-annotated, research outcomes from *Drosophila* or *Anopheles* insects may not be able to find a direct link in bees using sequence similarities. *De novo* studies are still required in many scenarios. In this Research Topic, McMenamin et al. identified a novel protein Bap1 involved in honey bee antiviral responses using transcriptomic methods and the Sindbis virus as a surrogate virus for *in vivo* infection studies. Bap1 is not universally present in insects; however, the most closely related orthology was discovered in cockroaches, which is intriguing since they are also susceptible to DWV infection (4) despite not being more closely related to the virus's usual insect hosts.

In summary, research articles on this Research Topic reported the findings of DWV subtypes and a novel antiviral protein Bap1. More novel antiviral proteins could soon be identified with the protocols for Bap1 identification, and the availability of laboratory-synthesized viral clones with fluorescence labels (5, 6). In addition, the emergence and evolution of DWV subtypes are yet to be fully understood. Whether subtype competition leads to stable levels is not yet known. It will be intriguing to find DWV subtype replacement events or the evolution of novel subtypes in the future.

## Author contributions

The author confirms being the sole contributor of this work and has approved it for publication.
